# Medaka: a promising model animal for comparative population genomics

**DOI:** 10.1186/1756-0500-2-88

**Published:** 2009-05-10

**Authors:** Yoshifumi Matsumoto, Hiroki Oota, Yoichi Asaoka, Hiroshi Nishina, Koji Watanabe, Janusz M Bujnicki, Shoji Oda, Shoji Kawamura, Hiroshi Mitani

**Affiliations:** 1 Department of Integrated Biosciences, Graduate School of Frontier Sciences, University of Tokyo, Tokyo, Japan; 2 Department of Developmental and Regenerative Biology, Medical Research Institute, Tokyo Medical and Dental University, Tokyo, Japan; 3 FUJIYA CO., LTD., Kanagawa, Japan; 4 Department of Medical Genome Sciences, Graduate School of Frontier Sciences, University of Tokyo, Tokyo, Japan; 5 International Institute of Molecular and Cell Biology, Warsaw, Poland; 6 Institute of Molecular Biology and Biotechnology, Faculty of Biology, Adam Mickiewicz University, Poznan, Poland; 7 Laboratory for Behavioral and Developmental Disorders, Brain Science Institute, RIKEN, Saitama, Japan

## Abstract

**Background:**

Within-species genome diversity has been best studied in humans. The international HapMap project has revealed a tremendous amount of single-nucleotide polymorphisms (SNPs) among humans, many of which show signals of positive selection during human evolution. In most of the cases, however, functional differences between the alleles remain experimentally unverified due to the inherent difficulty of human genetic studies. It would therefore be highly useful to have a vertebrate model with the following characteristics: (1) high within-species genetic diversity, (2) a variety of gene-manipulation protocols already developed, and (3) a completely sequenced genome. Medaka (*Oryzias latipes*) and its congeneric species, tiny fresh-water teleosts distributed broadly in East and Southeast Asia, meet these criteria.

**Findings:**

Using *Oryzias *species from 27 local populations, we conducted a simple screening of nonsynonymous SNPs for 11 genes with apparent orthology between medaka and humans. We found medaka SNPs for which the same sites in human orthologs are known to be highly differentiated among the HapMap populations. Importantly, some of these SNPs show signals of positive selection.

**Conclusion:**

These results indicate that medaka is a promising model system for comparative population genomics exploring the functional and adaptive significance of allelic differentiations.

## Background

The accumulation of human genetic polymorphism data provided by sources such as the international HapMap project [1,2] has revealed a number of SNP sites with markedly different allele frequencies among human populations. Such data make systematic searches for disease-causing or drug-responsive genomic regions possible [3,4], and the accumulated SNP data can also provide compelling evidence of positive selection during human evolution [5,6]. An inevitable issue, however, is that mutagenesis and/or crossing-over experiments to elucidate functional differences between alleles at these polymorphic sites are practically impossible in humans. A vertebrate model animal with a broad geographic distribution and documented high genetic polymorphism could serve as a "natural library" of genetic variation in humans for orthologous genes that could be under similar selective pressures.

The medaka (*Oryzias latipes*) is a notable candidate for such a model animal. This small freshwater fish is found in East Asia with closely related congeneric species broadly distributed throughout Southeast Asia, and it has a long history of use as an experimental animal since the early 20th century. A number of inbred medaka strains have been established, and transgenesis and mutagenesis protocols have been developed, suggesting that medaka has great potential for use in systematic genetic analyses [7-10]. Medaka genome sequences are also available [11]. The greatest advantage of using medaka is its enormous genetic diversity compared to the other fish models (zebrafish, pufferfish, etc.), with the average nucleotide difference of 3.4% between two inbred medaka strains being the highest among any vertebrates thus far documented [11]. In this study, our purpose is to assess the validity of medaka as a useful resource of comparative population genomics.

## Methods

### Medaka strains

Japanese medaka (*Oryzias latipes*) populations consist of four geographical populations. We selected 24 wild-type strains from the Japanese medaka (see Additional file 1) and three closely related congeneric species (*O. curvinotus*, *O. luzonensis *and *O. celebensis*; see Additional file 2). We also examined an inbred strain (Hd-rR) of Southern Japanese origin.

### PCR-direct sequence, mRNA extraction and cDNA sequence

**Figure 1:**
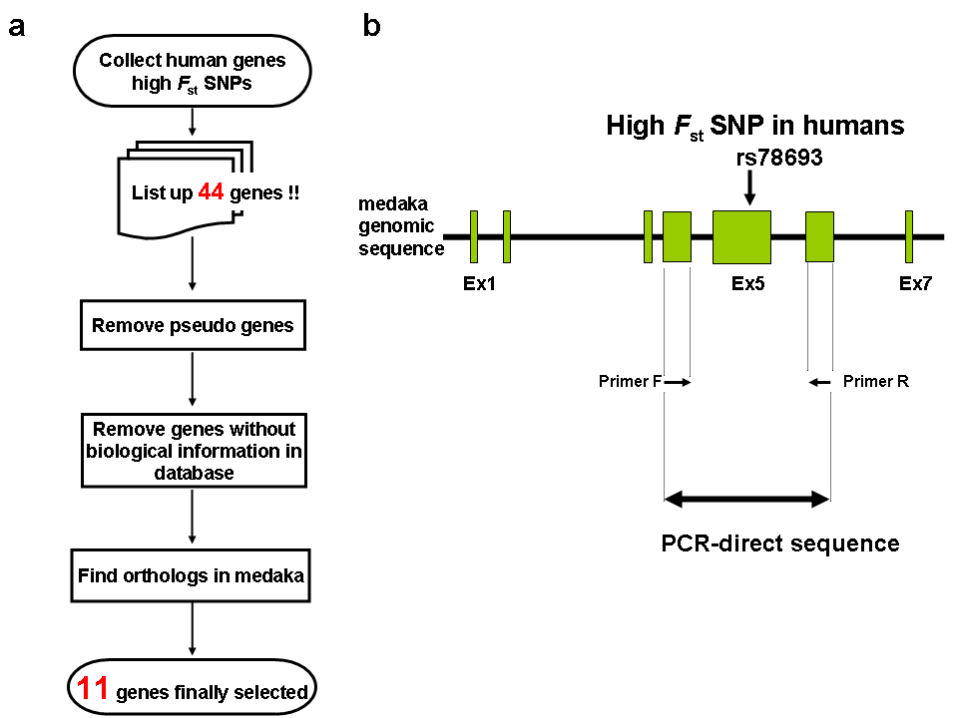
**(a) Flow chart of targeted gene selection, and (b) a schema of the SNP screening method**. We first focused on 44 genes with allele frequencies highly differentiated among human populations, including the 27 genes listed in Table nine of the first HapMap paper [1] based on high *F*_st _values for nonsynonymous SNPs and the 17 genes listed in Table S4 of Sabeti et al. (2006) in the category "population differentiation." A SNP site with a *F*_st _value higher than the genome average represents higher population differentiation at this site [26], possibly driven by natural selection [27-30]. Secondly, from the 44 genes, we removed pseudogenes, genes with unclear annotation and genes without biological information in the database. Thirdly, we chose genes for which only a single gene was assigned as an ortholog in the medaka genome by searching the Ensembl database . After applying these selection criteria, 11 genes were subjected to the SNP screening (Table 1).

We selected 11 genes for the screening of madaka SNPs (Table 1). The flow chart of the target gene selection is shown in Figure 1a. The PCR primers were designed on the basis of the medaka genomic sequences [11] corresponding to those of humans where high-*F*_st _SNPs are found (Fig. 1b). The PCR performed using genomic DNA extracted from medaka fins or bodys as template. To isolate entire *oRTTN *sequences, mRNAs were prepared from the embryos of seven strains, because we had already confirmed by in situ hybridization that *oRTTN *was expressed in early developmental stages. *oRTTN *genes were PCR amplified with primer pairs designed using the *oRTTN *sequence predicted from the medaka genome project (Hd-rR strain)[11]. The PCR products were directly sequenced using an ABI PRISM 3130-Avant Genetic Analyzer (Applied Biosystems Japan, Tokyo, Japan) and a total of ~340 kb of DNA sequences were obtained. The primer sequences and the determined sequences have been deposited in the international GenBank/DDBJ/EMBL nucleotide sequence database [accession nos. AB435679 – AB435956]. The thermocycling conditions are available on request.

**Table 1:** The 11 genes examined in this study

Gene	Gene ontology "biological process" annotation
*ALDH2*	alcohol metabolic process
*EDAR*	I-kappaB kinase/NF-kappaB cascade
*F2*	coagulation factor II
*GRK4*	regulation of G-protein coupled receptor protein signaling pathway
*LCT*	Lactase
*RTTN*	required for axial rotation and left-right specification
*SLC24A5*	solute carrier family 24, member 5
*SLC30A9*	solute carrier family 30 (zinc transporter), member 9
*SLC45A2*	solute carrier family 45, member 2
*LWS*	opsin 1 (cone pigments), long-wave-sensitive (color blindness, protan)
*THEA2*	response to temperature stimulus

### Statistical and phylogenetic analysis

Nucleotide sequences were aligned using CLUSTALW [12]. The pairwise *dN *and *dS *values among strains of 11 genes were calculated by DnaSP Software (version 4.0) according to the Nei-Gojobori method [13]. Insertions and deletions (indels) were excluded from analysis. For the entire nucleotide sequence of *RTTN*, the *d*N-*d*S and *p*-values were calculated by MEGA 4 [14] according to the Nei-Gojobori method with statistical significance tested by Z-tests.

### Protein structure prediction

The GeneSilico metaserver [15] was used to predict protein secondary structure and order/disorder, and to carry out fold-recognition (i.e. match the query sequence with structurally characterized templates). Potential phosphorylation sites were predicted using a semi-independent component of the metaserver available at the URL . For the THEA2 protein, the metaserver indicated very high similarity (PCONS score 3.28) of residues 1–360 (human numbering) to known Acyl-CoA hydrolase structures (e.g. 2gvh in the Protein Data Bank) and high similarity of residues 360–607 (PCONS score 2.00) to lipid transfer proteins from the STAR family (e.g. 1ln1 in the PDB). Long regions of intrinsic conformational disorder were predicted for loops connecting structural domains (around residues 160–200 and 340–370). For the RTTN protein, the metaserver identified the α-helical armadillo domain of β-catenin (1i7w in Protein Data Bank) as the best modeling template, in particular for residues 1–120, with a high confidence score (PCONS score 1.67). Long regions of structural disorder, devoid of secondary and tertiary structure, were predicted for residues 120–160 and 280–370. Three-dimensional structural models of the ordered (i.e. stably folded) parts of THEA2 and RTTN proteins were generated and optimized using the FRankenstein's Monster method [16]. The final models were evaluated as good quality by the PROQ server [17]. The models were expected to exhibit a root mean square deviation to the true structures in the order of 2–4 Å, suggesting that they are sufficiently reliable to make functional predictions at the level of individual amino acid residues. The atomic details of these models, however, must be taken with a grain of salt.

## Results and discussion

**Figure 2:**
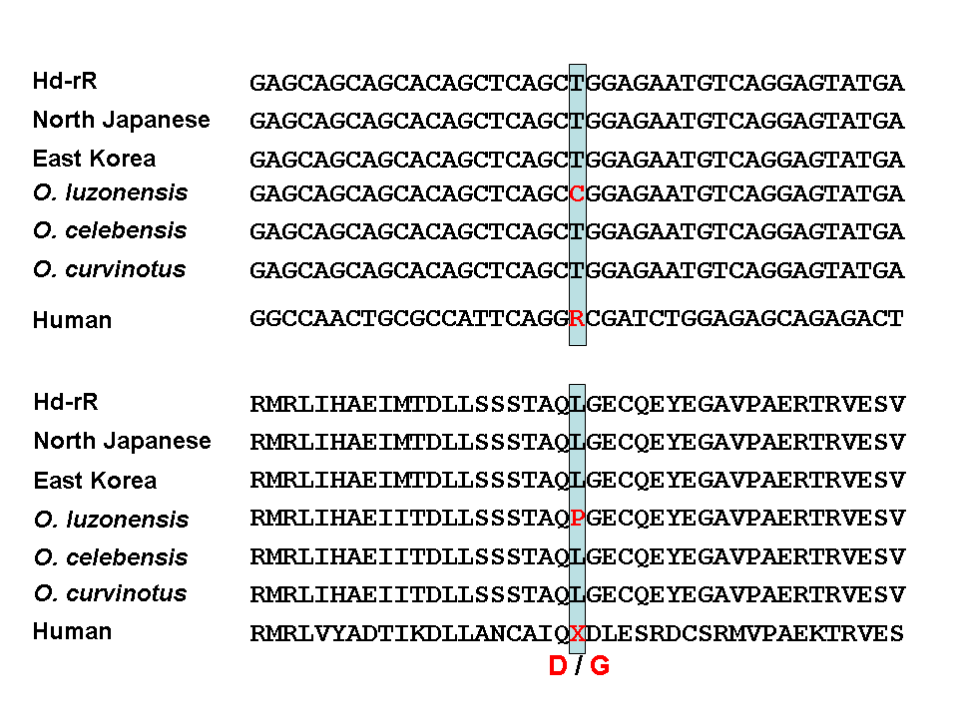
**Nucleotide (upper) and amino acid (lower) sequence alignments of *THEA2***. Hd-rR is the inbred strain derived from the southern Japanese population for which the complete genome sequence has been determined [11]. All three (Hd-rR, Northern Japanese and East Korea) are *Oryzias latipes*. The others are closely related species.

Of the 11 genes, we found that medaka *THEA2 *(*BFIT2*) contained a nonsynonymous SNP at the exactly same site where a high *F*_st _is observed in humans (rs1702003 in exon 6: see the HapMap database; Fig. 2). *THEA2 *is known to be a temperature responsive gene, and it is expressed in brown adipose tissue (BAT) in response to cold stress in mice [18]. The genotype frequencies at rs1702003 are 98.3% G/G and 1.7% G/A in Europeans and 100% A/A in East Asians and Africans. This could suggest that the European-specific allele of the cold-inducible gene is an adaptation of Europeans to the cold environment around 40,000 years ago when early modern humans expanded to Europe. Interestingly, only Philippine medaka (*Oryzias luzonensis*), inhabiting a warmer environment, has a different allele from the other *Oryzias *species. While in *situ *hybridization showed *THEA2 *is expressed ubiquitously in medaka embryos, RT-PCR indicated greater *THEA2 *expression in the brown tissue homologous to mammalian BAT than in the other tissues in adult medaka (data not shown). In the structural predictions for the THEA2, we found that the two SNPs indicated for the human and medaka proteins are located at the junction between the Acyl-CoA hydrolase structural domains in a loop predicted to be highly flexible. There, a G-D (in humans) or L-P change (in medaka) is likely to affect the dynamics of the protein chain and influence (1) the interaction between domains and/or (2) the transmission of conformational changes. We speculate that the amino acid change that affects protein flexibility may be related to temperature adaptation.

**Figure 3:**
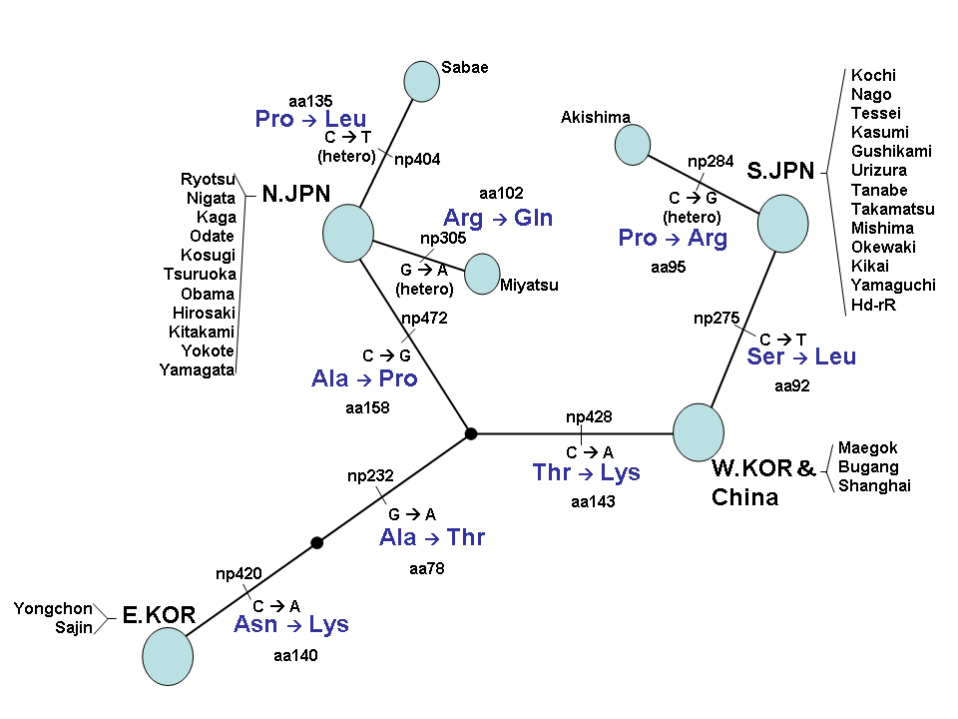
**Phylogenetic network of *RTTN *based on nucleotide sequences from exons 3 + 4 (271 bp)**. The circle represents geographical regional strains (N.JPN: northern Japanese population; S.JPN: southern Japanese population; W.KOR: western Korean; E.KOR: eastern Korean). "np" represents the nucleotide position number. The "aa" numbers are the amino acid sequence positions.

**Figure 4:**
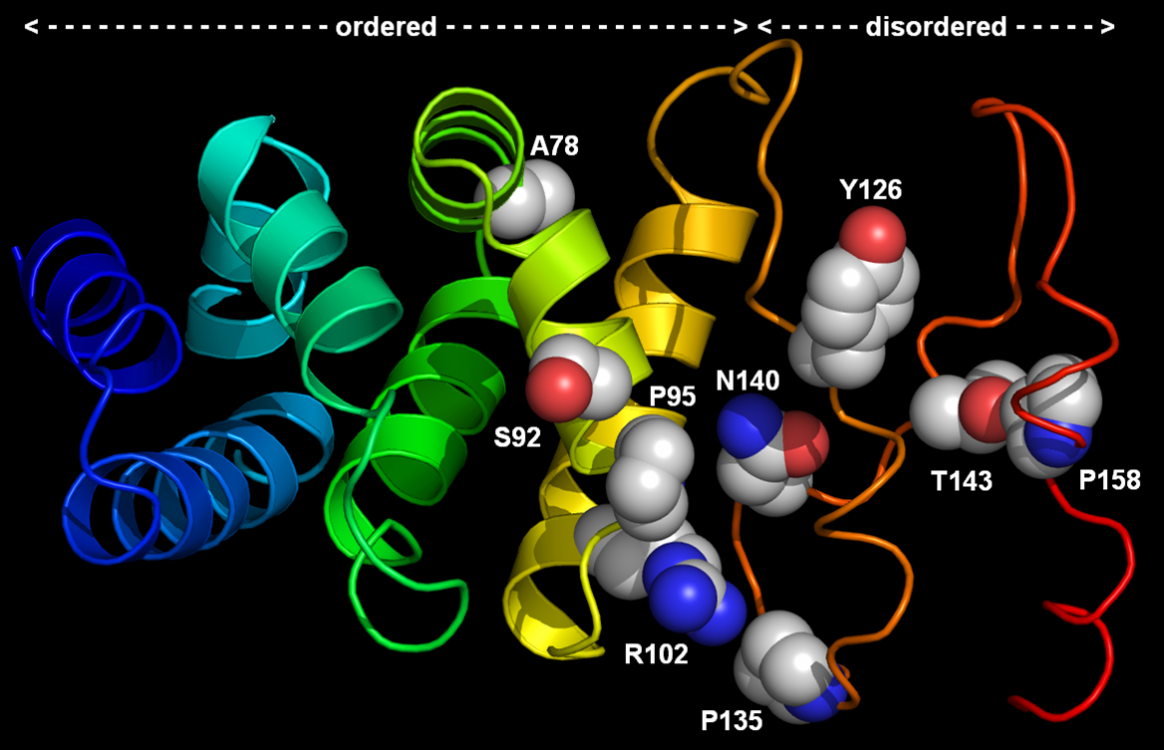
**Structure prediction for *RTTN*: a well-folded globular part (armadillo-like repeats, aa 1 – 120) and an unstructured linker (aa 121 – 166)**. The protein chain is colored from blue (N-terminus) to red (C-terminus). α-helices are shown as ribbons. Side chains of residues substituted because of SNPs are shown in the "spacefill" representation and labeled; C, O, and N atoms are shown in gray, red, and blue, respectively. The positions of amino acid changes and the medaka populations sharing these changes are as follows: aa78, Thr: E.KOR, Ala: Others; aa92, Ser: S.JPN, Leu: Others; aa95, Arg: Akishima, Pro: Others; aa102, Gln: Miyatsu, Arg: Others, aa135, Leu: Sabae, Pro: Others; aa140, Lys: E.KOR, Asn: Others; aa143, Thr: E.KOR, N.JPN, Lys: W.KOR & China, and S.JPN; aa158, Ala: N.JPN, Pro: Others.

For another gene, *RTTN*, we found even more remarkable regional differentiation. The phylogenetic network adding nine individuals from the northern Japanese population and one southern Japanese population indicates the nucleotide changes in the *RTTN *gene among geographical populations; each population forms a separate cluster and is separated by unique amino acid changes (Fig. 3). According to bioinformatic predictions, the RTTN protein is comprised of armadillo-like repeats separated in a few places by disordered loops (Fig. 4). A78 is partially buried and its substitution may destabilize the protein structure. S92 is located on the surface and is predicted to be phosphorylated; hence, its substitution may affect structure and/or function by removing a site of posttranslational modification. N140, T143, and P158 are in the disordered loop. Substituting P158 with A may increase the flexibility of the main chain, the introduction of K140 and K143 may increase the entropy of the side chain, and substitution of T143 (predicted to be phosphorylated) may remove a site of posttranslational modification. Thus, substitutions of all these residues are predicted to influence the dynamics of the loop and thus its ability to bind to other molecules or to respond to changes in the environment.

**Figure 5:**
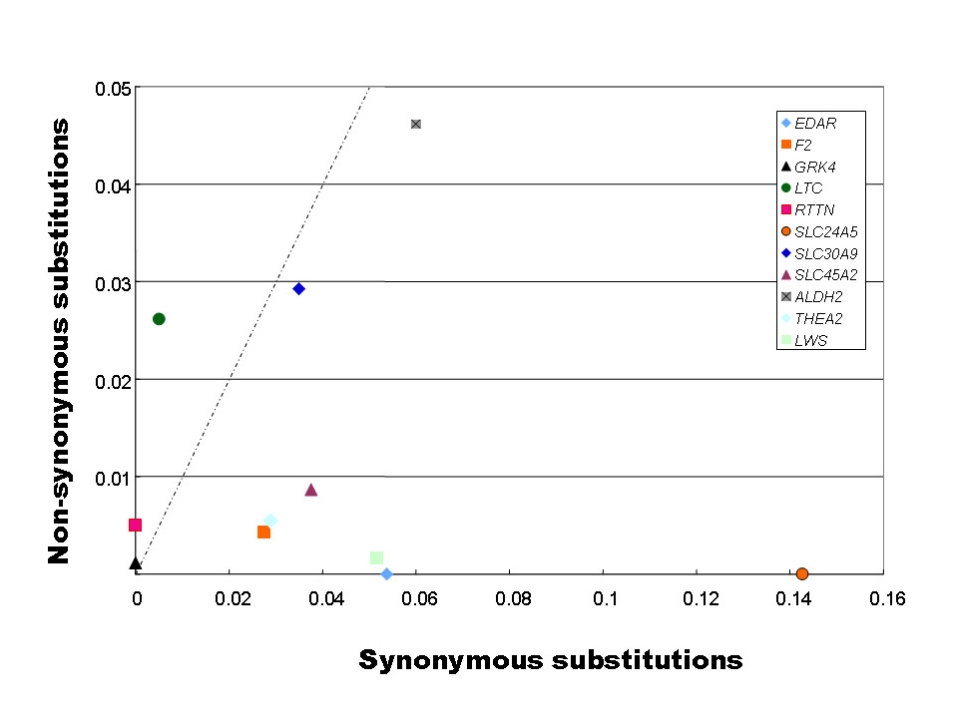
**Synonymous (X axis) and nonsynonymous (Y axis) substitution ratios estimated by the Nei – Gojobori method**. A *d*N/*d*S ratio significantly greater than 1 is a convincing indicator of positive selection.

To gain further insight into whether natural selection is involved in the observed nucleotide variations, we plotted the average number of nonsynonymous nucleotide differences per number of nonsynonymous sites (*d*_*N*_) against the average number of synonymous nucleotide differences per number of synonymous sites (*d*_*S*_) estimated for the 11 genes among the 27 medaka strains (Fig. 5). Seven of the 11 genes including *THEA2 *showed an average *d*_N_/*d*_S _of less than 1, suggesting that the seven genes are under purifying selection. In *RTTN*, in contrast, there are only nonsynonymous differences in the genomic regions examined (exons 3 and 4: 271 bp in total); in more than half of the population pairs, the *d*_N_/*d*_S _ratios are significantly greater than 1 (Z-test; *p *< 0.05). The *d*_N_/*d*_S _ratios of the *LTC *and the *GRK4 *genes are also greater than 1, but these are not statistically significant at 5% level for any pair. We have sequenced the entire *RTTN *cDNA for seven individual medaka from five geographical populations. Although there are synonymous variations in the other exons, the *d*_N_/*d*_S _ratios are overall greater than 1, and in nine of the 21 pairs they are statistically significant (*p *< 0.05; Table 2). These results suggest that *RTTN *is under positive selection in medaka.

**Table 2:** The *d*N - *d*S values (upper diagonal) and the significance (lower diagonal) based on *RTTN *cDNA (5.8 kb) sequences

	**Population samples**	**Nigata **	**Iwaki **	**Mishima**	**Nago**	**Shanghai**	**Maegok**	**Yongchon**
N.JPN	Nigata		1.622	1.712	1.685	1.116	1.835	1.87
	Iwaki	0.054		2.988	1.674	0.847	1.567	1.077
S.JPN	Mishima	0.045	0.002*		2.005	0.91	1.507	1.072
	Nago	0.047*	0.048*	0.024*		1.009	1.886	0.876
China	Shanghai	0.133	0.199	0.182	0.157		0.549	1.218
W.KOR	Maegok	0.035*	0.06	0.067	0.031*	0.292		2.103
E.KOR	Yongchon	0.032*	0.142	0.143	0.191	0.113	0.019*	

* represents 5% level significance of *p*-value in Z-test. "N. JPN" and "S.JPN" represent North and South Japan medaka, respectively. "W.KOR" and "E.KOR" represent West and East Korea medaka, respectively

Although its exact function is not known, *RTTN *is reported to be involved in determining the rotation of the body axis and the left-right asymmetry of internal organs during the embryonic development of mice [19]. The conspicuous differentiation of *RTTN *alleles among human populations also suggests differential natural selection acting on different populations: at a nonsynonymous SNP site (rs3911730) in the *RTTN *exon 3, the A/A genotype occurs in 90% of Africans, 2% of Europeans and is absent in Asians, while the C/C genotype occurs in 3% of Africans, 80% of Europeans and 100% of Asians.

Previous studies have reported that genes identified in fish through "forward genetic" analysis of phenotypic mutants are involved in forming variations of related phenotypes in humans, e.g. of skin pigmentation [20-24] and epithelial development [25]. Our approach in this study is an extension of these previous studies, as a form of "reverse genetics" of genes that show, as a signature of natural selection acting on them, a prominent level of diversification in the allele frequency among populations with different ecological histories in both fish and humans. We found that out of 11 genes in our analysis, the medaka *THEA2 *gene has a nonsynonymous polymorphic site at exactly the same position as its ortholog in humans, and the *RTTN *gene shows signs of population differentiation that can be explained plausibly by natural selection. The aim of our analysis is not to demonstrate evidence of natural selection in medaka, but to indicate that medaka is a marvelous resource as a "natural library" of genetic diversity, and this approach is efficient enough to find candidate genes targeted by natural selection in both humans and medaka. The exact function of the genes and the exact nature of the functional differences between alleles can be studied more feasibly in medaka, where crossing experiments between different genotypes of interest and transgenic techniques have already been established [7,8]. This method can be applied to any polymorphic gene in humans, and larger-scale and more systematic screening of orthologous gene polymorphisms in medaka will find various target genes for further functional analyses. As the medaka has been widely used for carcinogenesis and ecotoxicological studies [7], for example, in screening for genetic variants concerning medaka carcinogenesis and ecotoxins, it could also be used for testing variations in drug response in humans. Thus, we conclude that the medaka is a good vertebrate model of the functional diversity caused by human DNA polymorphisms that have been identified by recent resequencing and typing efforts.

## Authors' contributions

HO conceived, and SK and HM formed the project. SO and HM provided the medaka resources. HO, YM, and HM designed the experiments. KW and YM performed PCRs and sequencing. For *THEA2 *and *RTTN*, YM performed the RT-PCRs and cDNA sequencing, YA and HN performed WISH. JMB performed protein structure predictions. HO and YM analyzed the data and wrote the paper. All authors read and approved the final manuscript.

## Supplementary Material

Additional file 1 **Sampling map of regional strains for *Oryzias latipes***. Four strains (Nigata, Ryotsu, Kaga and Odate) are from the Northern Japanese population, and 15 strains (Tanabe, Takamatsu, Tessei, Kasumi, Uridura, Iwaki, Mishima, Hagi, Okewaki, Kikai, Nago, Kochi, Yamaguchi, Akishima and Gushikami) are from the Southern Japanese population. Two strains (Yongchon and Sajin) are from the Eastern Korean population, and three strains (Maegok, Bugang and Shanghai) are from Western Korean and Chinese populations. For the *RTTN *gene, we examined nine additional individuals from seven wild strains (Kosugi, Tsuruoka, Obama, Hirosaki, Kamikita, Yokote, Yamagata) from the Northern Japanese population. Click here for file

Additional file 2 **Sampling map of regional strains for closely related species.** Click here for file
