# Supplementary figures and images for: Medaka: a promising model animal for comparative population genomics
Source: BMC Res Notes. 2009 May 10;2:88. doi: 10.1186/1756-0500-2-88 (PMC2683866; doi:10.1186/1756-0500-2-88)

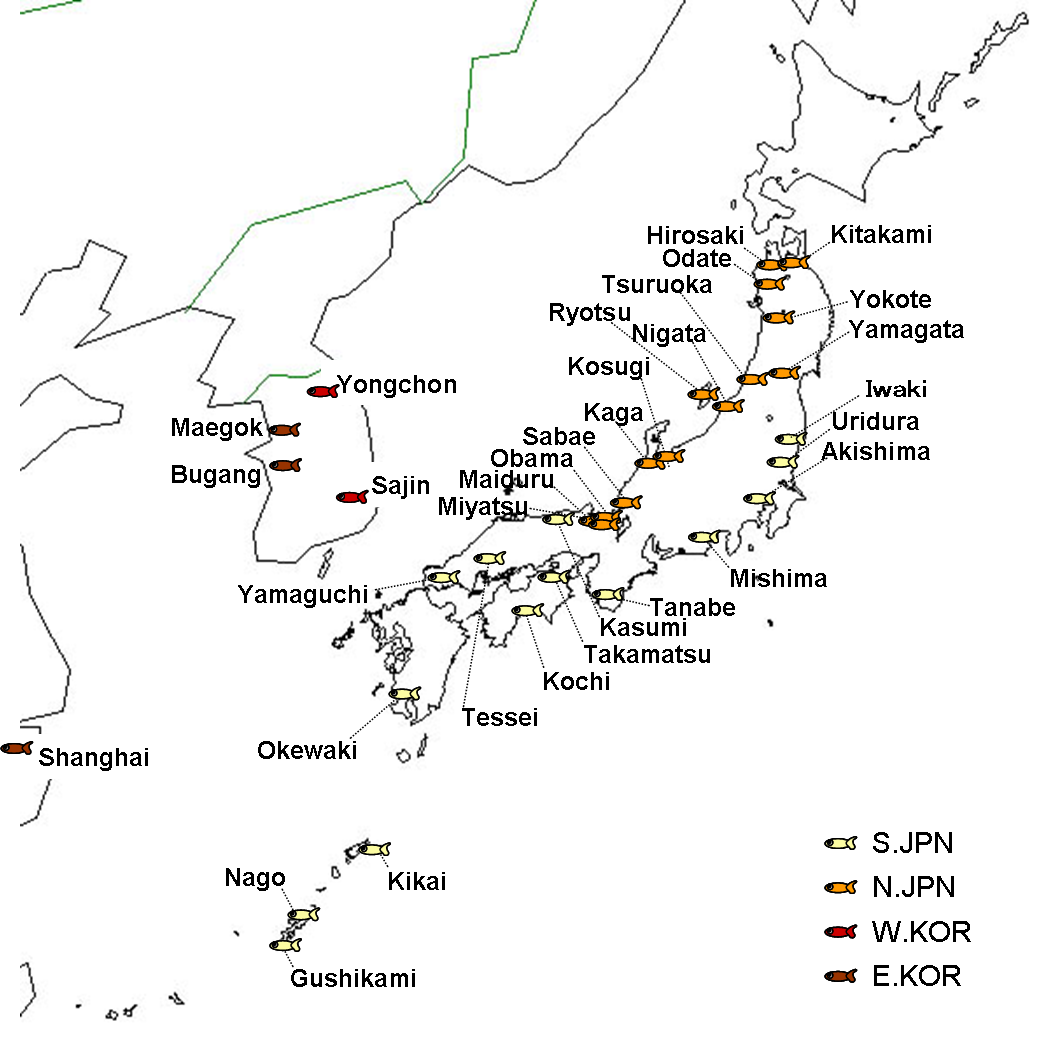

Supplement: Additional file 1 — Sampling map of regional strains for Oryzias latipes. Four strains (Nigata, Ryotsu, Kaga and Odate) are from the Northern Japanese population, and 15 strains (Tanabe, Takamatsu, Tessei, Kasumi, Uridura, Iwaki, Mishima, Hagi, Okewaki, Kikai, Nago, Kochi, Yamaguchi, Akishima and Gushikami) are from the Southern Japanese population. Two strains (Yongchon and Sajin) are from the Eastern Korean population, and three strains (Maegok, Bugang and Shanghai) are from Western Korean and Chinese populations. For the RTTN gene, we examined nine additional individuals from seven wild strains (Kosugi, Tsuruoka, Obama, Hirosaki, Kamikita, Yokote, Yamagata) from the Northern Japanese population. [file 1756-0500-2-88-S1.tiff]

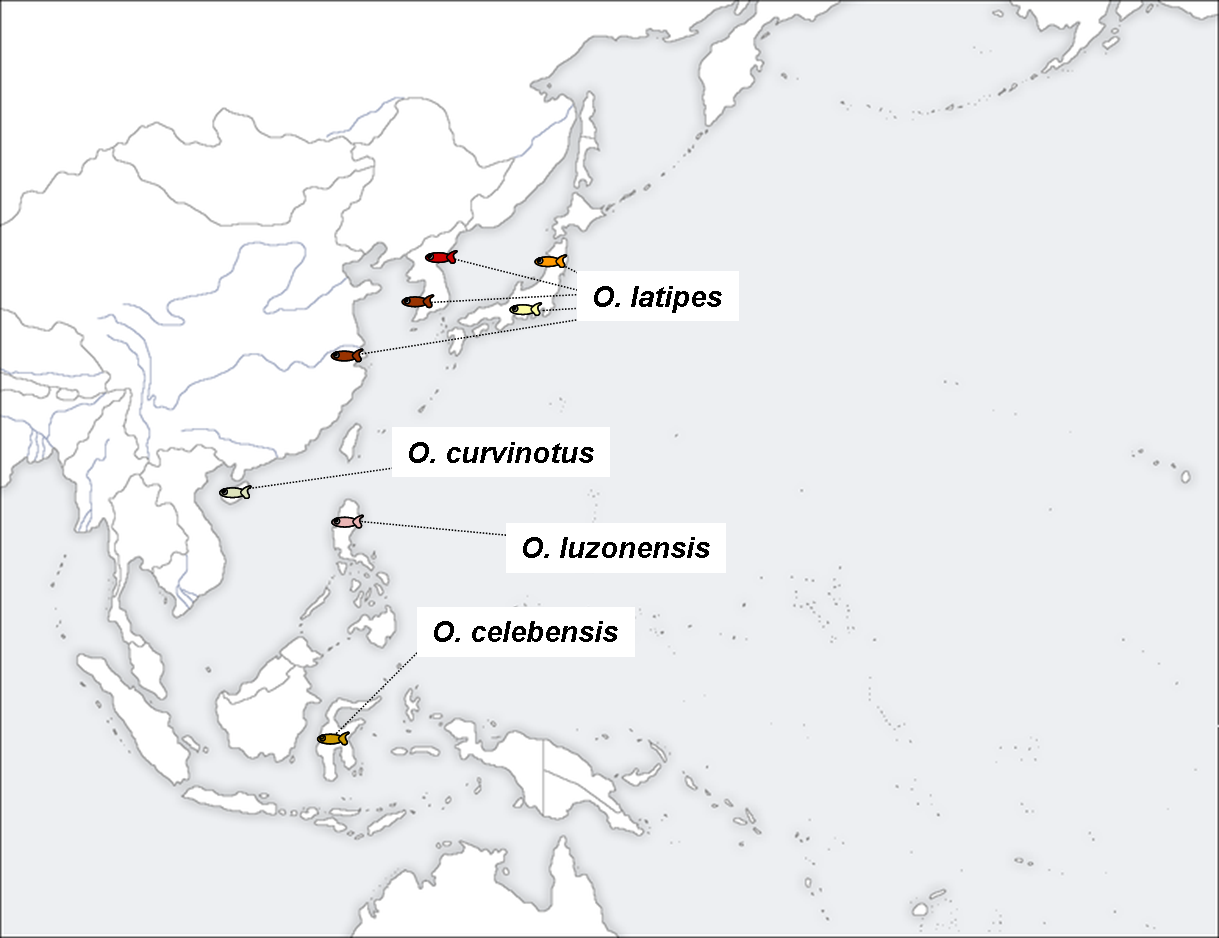

Supplement: Additional file 2 — Sampling map of regional strains for closely related species. [file 1756-0500-2-88-S2.tiff]
